# *Salmonella* Phage vB_SpuM_X5: A Novel Approach to Reducing *Salmonella* Biofilms with Implications for Food Safety

**DOI:** 10.3390/microorganisms12122400

**Published:** 2024-11-22

**Authors:** Xinxin Jin, Xiuxiu Sun, Qin Lu, Zui Wang, Zhenggang Zhang, Xiaochun Ling, Yunpeng Xu, Ruiqin Liang, Junjie Yang, Li Li, Tengfei Zhang, Qingping Luo, Guofu Cheng

**Affiliations:** 1 College of Veterinary Medicine, Huazhong Agricultural University, Wuhan 430070, China; 2 Key Laboratory of Prevention and Control Agents for Animal Bacteriosis (Ministry of Agriculture and Rural Affairs), Institute of Animal Husbandry and Veterinary, Hubei Academy of Agricultural Sciences, Wuhan 430064, China; 3 Hubei Provincial Key Laboratory of Animal Pathogenic Microbiology, Institute of Animal Husbandry and Veterinary, Hubei Academy of Agricultural Sciences, Wuhan 430064, China; 4 Hubei Hongshan Laboratory, Wuhan 430070, China

**Keywords:** *Salmonella*, phage, biofilm, bacteriostatic agent

## Abstract

*Salmonella*, a prevalent foodborne pathogen, poses a significant social and economic strain on both food safety and public health. The application of phages in the control of foodborne pathogens represents an emerging research area. In this study, *Salmonella pullorum* phage vB_SpuM_X5 (phage X5) was isolated from chicken farm sewage samples. The results revealed that phage X5 is a novel *Myoviridae* phage. Phage X5 has adequate temperature tolerance (28 °C–60 °C), pH stability (4–12), and a broad host range of *Salmonella* bacteria (87.50% of tested strains). The addition of phage X5 (MOI of 100 and 1000) to milk inoculated with *Salmonella* reduced the number of *Salmonella* by 0.72 to 0.93 log_10_ CFU/mL and 0.66 to 1.06 log_10_ CFU/mL at 4 °C and 25 °C, respectively. The addition of phage X5 (MOI of 100 and 1000) to chicken breast inoculated with *Salmonella* reduced bacterial numbers by 1.13 to 2.42 log_10_ CFU/mL and 0.81 to 1.25 log_10_ CFU/mL at 4 °C and 25 °C, respectively. Phage X5 has bactericidal activity against *Salmonella* and can be used as a potential biological bacteriostatic agent to remove mature biofilms of *Salmonella* or for the prevention and control of *Salmonella*.

## 1. Introduction

*Salmonella* is a common foodborne pathogen that poses a risk to public safety [1]. It is a zoonotic pathogen that causes foodborne outbreaks and epidemics of varying degrees worldwide [2,3]. *Salmonella* is responsible for 80% to 90% of food-borne bacterial poisonings in China [4]. In Columbia and Puerto Rico, a total of 1135 individuals became ill due to consuming poultry contaminated with *Salmonella* in the 2021. Among them, 2 people died and 273 were hospitalized [5]. *Salmonella pullorum* is highly infectious in chicks under three weeks of age, with morbidity and mortality rates reaching up to 100%. This pathogen significantly impacts the performance and intestinal health of poultry, leading to severe economic losses for the poultry industry [6,7]. Currently, the best strategy to reduce pullorosis is to eliminate *S. pullorum* in chicken farms [8]. Chickens infected with *Salmonella* represent the main source of *Salmonella* infection in humans [9]. *Salmonella* usually contaminates a variety of foods such as milk, eggs, and meat.

Phages or bacteriophages are viruses that invade bacteria. Phages, which are avirulent, specific, and sensitive to drug-resistant bacteria [10,11], have been seldomly studied as a bacteriostatic agent to prevent pathogens from contaminating foods and to ensure food safety [12,13]. The phage mixture provides reference for the research and application of phages against *Salmonella* infection in chicken [14]. Bacteria in biofilms are not affected by antibiotics. As a result, they are difficult to treat. Few studies have demonstrated the effectiveness of phages and their derivatives in inhibiting *S. typhimurium* biofilms [15]. However, there is limited information on the application of phages to remove mature biofilms of *S. pullorum* and reduce *S. pullorum* contamination in chicken breasts in China. To the best of our knowledge, this is the first study that evaluates the effect of phages on the biofilm of *S. pullorum*, providing some reference for the control of the bacteria.

In this study, *Salmonella* phage X5 was isolated from sewage samples obtained from a chicken farm. The morphological and biological characteristics of the phage were determined, and the effect of the phage on the biofilm formation of *Salmonella* was evaluated. In addition, the bacteriostatic activity of the phage in milk and chicken breast meat was studied, which provided a theoretical basis for the development of bacteriostatic agents in foods.

## 2. Materials and Methods

### 2.1. Bacterial Strains and Media

*S. pullorum* 519 (CVCC 519) was purchased from the China Veterinary Culture Collection Center (CVCC, Beijing, China). The study used seven different strains of tested bacteria, including 80 *Salmonella* strains containing 3 different serotypes and 4 non-*Salmonella* strains (Table 1). These strains were stored at the Laboratory of Animal Pathology, College of Veterinary Medicine, Huazhong Agricultural University. Luria–Bertani (LB) broth and LB nutrient agar medium (Qingdao Haibo Biotechnology Company, Qingdao, China) were used for bacterial culture.

### 2.2. Isolation, Identification and Purification of Phage X5

Chicken feces and sewage samples were collected from a large-scale chicken farm in Hubei Province. Phage isolation was performed as previously described [16]. Briefly, all samples were centrifuged at 11,000× *g* for 15 min to remove large particulate matter and were then passed through a 0.22-μm filter (Millipore, Burlington, MA, USA) to remove bacterial cells and small impurities. Phage enrichment was achieved by mixing 0.1 mL of *S. pullorum* 519 solution with 1 mL of filtrate and 10 mL of LB broth. The mixture was cultured overnight in a 37 °C incubator, centrifuged at 11,000× *g* for 15 min, and passed through a 0.22-μm filter. The traditional double-layer agar (DLA) technique was used to verify the presence of phages [17]. DLA plates were incubated overnight at 37 °C. Positive samples were those with transparent plaques. Clear plaques were selected and purified five times to obtain purified phages. The phages were stored at 4 °C prior to use and stored at −80 °C with 30% glycerol for long-term storage.

### 2.3. Determination of Host Range of Phage X5

The host range of phage X5 was determined using the dropping method [18]. Except for *Clostridium perfringens* and *Lactobacillus acidophilus*, the transparent lawns of the tested strains were formulated with LB nutrient agar. The lawns of *C. perfringens* were prepared using TSC medium supplemented with 0.05% D-cycloserine, and those of *L. acidophilus* were prepared using MRS medium with 1.5% agar based on 42 °C. A phage suspension of 5 μL (10^8^ PFU/mL) was dropped on the lawns of the strain. The plates were incubated overnight at 37 °C to form transparent lytic plaques. Plaque formation confirms the host’s susceptibility to phages. The phage host range of each test strain was assessed using a scoring system [19]. The experiment was repeated three times.

### 2.4. Determination of Biological Characteristics of Phage X5 

Phage X5 was incubated under different conditions to determine its tolerance to various environments. The purified phage samples (1 mL phage, 10^10^ PFU/mL) were incubated at 28, 37, 50, 60, and 70 °C to determine the thermal stability, and the titers were determined by the DLA method at 20, 40, and 60 min [20]. To assess pH tolerance, 100 μL phage suspension (10^10^ PFU/mL) was mixed with 900 μL fresh LB broth at different pH values (pH 2–12, adjusted with HCl or NaOH). After oscillating the phage suspensions at 37 °C for 1 h (160 rpm/min), the phage titer was determined using the DLA technique [21,22].

According to the infection ratio of 1000, 100, 10, 1, 0.1, 0.01, and 0.001, the mixture of *Salmonella* CVCC519 and phage X5 was supplemented with LB broth, so that the final volume of each tube was the same. Each treatment had three replicates. The phage titer was determined by the DLA method in a shaker (150 rpm) at 37 °C for 4 h [23]. Chloroform was mixed with phage X5 at 0, 25, and 50% [24]. Each mixture was incubated at 220 rpm for 1 h at 37 °C. Phage titer was determined by the DLA technique. The experiments were carried out in triplicate.

### 2.5. Adsorption Rate Determination

In this experiment, 3 mL of the logarithmic growth stage of *S. pullorum* (CVCC 519) was transferred into a centrifuge tube. Subsequently, phage X5 (3 mL) was added according to the optimal multiplicity of infection (MOI; 0.01). The mixture was placed on a shaking table at 37 °C (150 rpm/min) and incubated for 50 min. We then removed 200 μL of the mixture every 5 min and placed it on ice for 1 min. The mixture was centrifuged at 12,000 rpm/min for 1 min, and the phage titer in the supernatant was determined using the DLA technique. The experiment was repeated three times. The phage adsorption rate was calculated using the following formula:

Phage adsorption rate = [(total number of initial phages − number of unadsorbed phages)/total number of initial phages] × 100% [25].

### 2.6. One–Step Growth Curve of Phage X5

One–step growth curve tests were performed as previously reported [26]. The phage lysate with a MOI of 0.01 and its host bacteria were added to LB broth medium and incubated at 37 °C for 10 min. The mixed medium was centrifuged at 12,000 rpm for 8 min, the supernatant was discarded, and the precipitate was washed three times with LB broth medium. Subsequently, 10 mL of preheated LB liquid medium at 37 °C was added to the precipitate and thoroughly mixed. The culture was mixed by oscillation at 160 rpm at 37 °C. Samples (100 μL) were collected every 10 min for 120 min and diluted in LB broth to determine the phage titer. The filtrate titer was determined by the DLA technique. The latency period is defined as the shortest time required for the phage to adsorb to the host bacteria and release progeny phages. The phage burst size represents the late phage burst titer divided by the concentration of host bacteria at the initial infection stage [27]. The experiment was repeated three times.

### 2.7. Transmission Electron Microscopy (TEM) of Phage X5

Freshly prepared phages (10^10^ PFU/mL) were centrifuged at 45,000× *g* for 2 h at 4 °C and precipitated with 500 μL of 0.1 M ammonium acetate. The phage drops were placed on a mesh for 10 min and stained with 2% (*w*/*v*) phosphotungstic acid for 15 min. The stained phages were photographed by TEM (Hitachi H–7000FA, Tokyo, Japan) at 75 kV [28]. The head diameter and tail length of phage X5 were measured using Digital Micrograph Demo 3.9.1 (Gatan, Inc., Pleasanton, CA, USA).

### 2.8. Phage X5 DNA Extraction and Genome Sequence Analysis

The extraction of phage X5 genomic DNA was performed as previously reported [29] and sequenced in an Illumina HiSeq platform (Illumina Inc., San Diego, CA, USA) [30]. Assembly of the reads was performed using SPAdes (version 3.12.0) software [31].

GeneMarkS (http://exon.gatech.edu/GeneMark/, accessed on 5 May 2024) and RAST (https://rast.nmpdr.org/rast.cgi, accessed on 5 May 2024) were used to predict the genome sequence of phage X5. The phage X5 genome map was generated by CGviewServer (http://cgview.ca/ accessed on 7 May 2024) [32]. The comprehensive antibiotic resistance database (CARD, http://arpcard.mcmaster.ca, accessed on 10 May 2024) and virulence factor database (VFDB, http://www.mgc.ac.cn/VFs/, accessed on 10 May 2024) were used to screen antibiotic resistance and virulence factor–related genes, respectively. A visual genome map of phage X5 was generated using an online website (https://cgview.ca/, accessed on 15 May 2024). The BLASTn webline website (https://blast.ncbi.nlm.nih.gov/Blast.cgi, accessed on 20 May 2024) was used to compare the nucleotide sequences of phages and search for highly similar phages in GenBank. BLASTP was used to search the NR (Non–Redundant) protein database and identify the functional annotation of CDS (Coding sequence) [33,34]. The functions of open reading frames (ORFs) were annotated using BLASTP and the Rast database. Using the virus classification of the International Committee on Taxonomy of Viruses and BLASTn of NCBI database (https://blast.ncbi.nlm.nih.gov/Blast.cgi, accessed on 20 May 2024), their terminal enzyme large subunits were selected to show significant similarity. MEGA 10 was used for phylogenetic and evolutionary analyses [35]. Visual genome comparison of phage X5 and GSP044 was performed using Mauve 2.3.1 [36]. The whole genome sequence of phage X5 has been uploaded to NCBI database (GenBank number: PP502935).

### 2.9. Ability of Phage X5 to Reduce Salmonella Biofilm Formation

To evaluate the impact of phage X5 on biofilm formation, this study referred to previous research and made slight modifications to the methodology [37,38,39]. First, the concentration of fresh CVCC519 was adjusted to OD600 = 0.5 (about 1 × 10^9^ CFU/mL). Second, 1 mL bacterial solution was added to each well of the 24–well cell culture plate, followed by 1 mL LB medium. Third, following three days of incubation at 37 °C, the medium was discarded, the wells were washed with 1 mL sterile phosphate buffered saline (PBS) three times, and phage X5 (10^5^ PFU and 10^7^ PFU) was added. Fourth, after incubation at 37 °C for 6 h, the media were discarded and washed three times with sterile PBS. Fifth, the bacterial load in the biofilm was measured. For the biofilm determination assay, following the addition of 1 mL of 95% methanol per well and incubation for 30 min, the fixed solution was discarded, and each well was washed three times with PBS. Subsequently, 1 mL crystal violet solution (1%) was added, and the plate was incubated for 30 min at room temperature. After washing with PBS three times, 1 mL of anhydrous ethanol was added for decolorization. Absorbance was measured at OD600. The bacteria were incubated, and the phage was replaced with an equal amount of SM buffer (Tris/NaCl/MgSO_4_), stained with crystal violet, and decolorized with ethanol. Absorbance was measured at OD600. Three parallel samples were set up for each experiment, and the experiments were repeated three times.

### 2.10. Inhibitory Effect of Phage X5 on Salmonella in Foods

The effects of the phage on *Salmonella* in different food substrates were evaluated in raw chicken breast and milk from a supermarket in Wuhan City.

Milk was confirmed to be aseptic by plate coating. Milk (9.8 mL) was mixed with 100 μL phage X5 (10^8^ or 10^9^ PFU/mL) and inoculated with 100 μL *S. pullorum* 519 (10^6^ CFU/mL, PBS suspension). The control groups consisted of 200 μL PBS. The mixtures were incubated at 25 °C and 4 °C. Samples were collected at 0, 2, 4, 6, 8, 18, and 24 h, and the recoverable bacteria in milk were detected by the *Salmonella Shigella* (SS) Agar plate counting method [40,41].

The raw chicken breasts were divided into small pieces (2 × 2 cm), placed on a clean workbench, and irradiated by UV for 20 min to ensure disinfection and sterilization. then, 100 μL log–growth *Salmonella* CVCC 519 (10^6^ CFU/mL) was added to each experimental sample and incubated at room temperature for 15 min. Subsequently, 100 μL of the phage (10^8^ PFU/mL, MOI = 100 and 10^9^ PFU/mL, MOI = 1000) was added, while an equal amount of sterile saline was added as a negative control. The treated samples were incubated at 4 °C for 0, 2, 4, 8, 18, and 24 h. Each piece of chicken was cut with sterile scissors and added to 1 mL sterile normal saline and mixed by oscillation for 3 min. The amount of *Salmonella* on the chicken pieces was determined by the dilution coated plate counting method [16].

### 2.11. Statistical Analysis

Data were statistically analyzed using GraphPad Prism 8.0.1 (San Diego, CA, USA). Results are presented as mean ± standard deviation (SD), and significance analysis between groups was performed using one–way ANOVA. Statistical significance was set at *p* < 0.05.

## 3. Results

### 3.1. Isolation and Identification of Salmonella Phage

**Figure 1:**
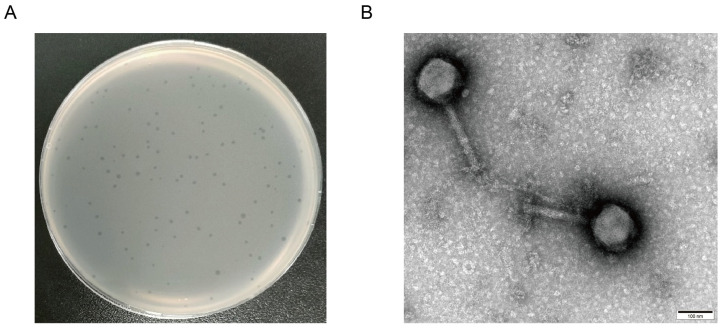
Morphology and microscopic morphology of phage X5 plaque. (**A**) Phage X5 plaque morphology. (**B**) Microscopic morphology of phage X5 under transmission electron microscopy. The scale is 100 nm.

A strain of *Salmonella* phage was isolated from the sewage samples of a chicken farm. The morphology and size of the plaque were similar (Figure 1A). The plaque, which was transparent and had no halo, was labeled vB_SpuM_X5 (X5). Phage X5 had a typical icosahedral structure with a head diameter of about 130 nm, a tail diameter of about 210 nm, and a retractable tail, consistent with the characteristics of Myoviridae (Figure 1B).

### 3.2. Host Range of Phage X5

Three serotypes of *Salmonella* were used to test the host range of phage X5. Host range analysis showed that phage X5 could form a transparent phagocytic circle when co–cultured with 70 strains of *Salmonella*. The cleavage rate reached 87.50% (70/80). However, phage X5 could not infect other genera of bacteria (Table 1). Additionally, phage X5 could not infect all the Gram–negative bacteria tested (*Klebsiella pneumoniae* and *Escherichia coli*) or two of the Gram–positive strains (*Lactobacillus acidophilus* and *Clostridium perfringens*).

### 3.3. Biological Characteristics of Phage X5

**Figure 2:**
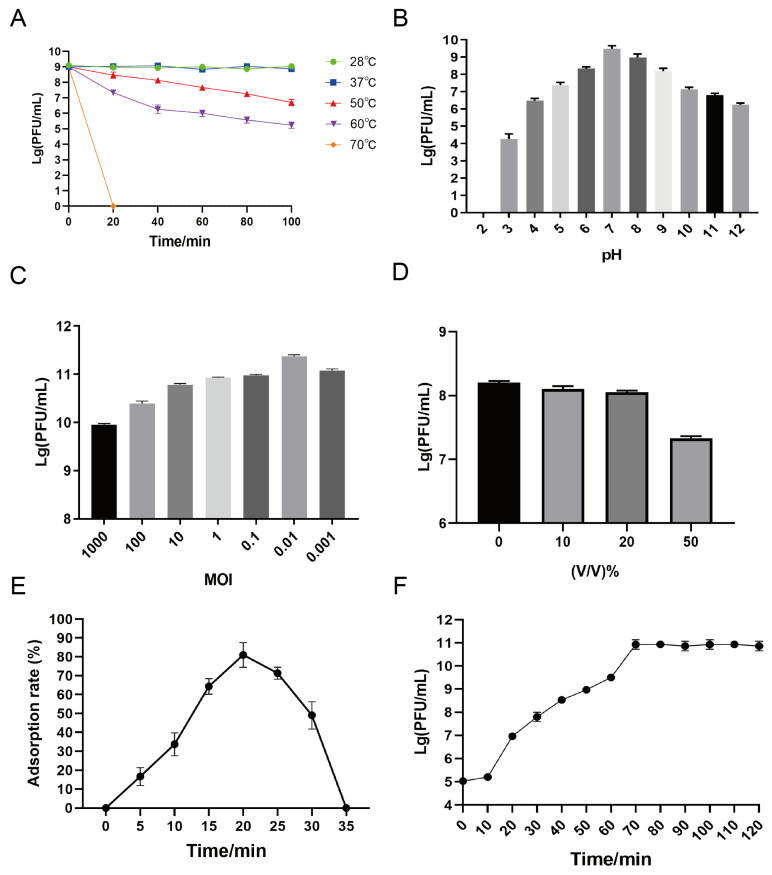
Biological characteristics of phage X5. Stability of phage X5 at different temperatures (**A**) and pH values (**B**). (**C**) Optimal MOI of phage X5. (**D**) Determination of chloroform sensitivity of phage X5. (**E**) Adsorption rate of phage X5. (**F**) One–step growth curve.

Figure 2 shows the biological characteristics of phage X5. The initial titer of phage X5 was 10^10^ PFU/mL. The titer of phage X5 was stable at 28 °C and 37 °C (Figure 2A). When the temperature was raised from 50 °C to 60 °C, the titer of the phage decreased and subsequently stabilized. The titers dropped sharply from 0 to 20 min, and the rate of the decline decreased from 20 to 60 min, whereas the phages were completely inactivated after 20 min at 70 °C. Figure 2B shows that phage X5 remained active and stable at pH 4–12; however, its stability decreased sharply and its titer could not detected at pH 2. Figure 2C shows the optimum MOI of phage X5. Phage X5 had the highest titer at MOI = 0.01; hence, the optimal MOI of phage X5 was 0.01, indicating that a small quantity of phages has the potential to lyse a significant number of bacteria, resulting in optimal titers.

Figure 2D shows the results of the chloroform sensitivity test. At ≤25% chloroform, the phage X5 titer was stable and high. At 50% chloroform, phage X5 was significantly reduced, but not completely inactivated.

### 3.4. Adsorption Rate

The adsorption rate of phage X5 increased from 0 to 20 min and reached its peak at 20 min with an adsorption rate of 81.0%. After 20 min, the adsorption rate began to decline (Figure 2E).

### 3.5. One–Step Growth Curve

Figure 2F shows a one–step growth curve of phage X5 at MOI = 0.01. The latent period was 10 min, the lysis period was 10–70 min, and the number of phages increased rapidly. In addition, the mean burst size of phage X5 was estimated to be 120 PFU/cell.

### 3.6. Whole–Genome Sequencing and Coding Gene Prediction of Phage X5

**Figure 3:**
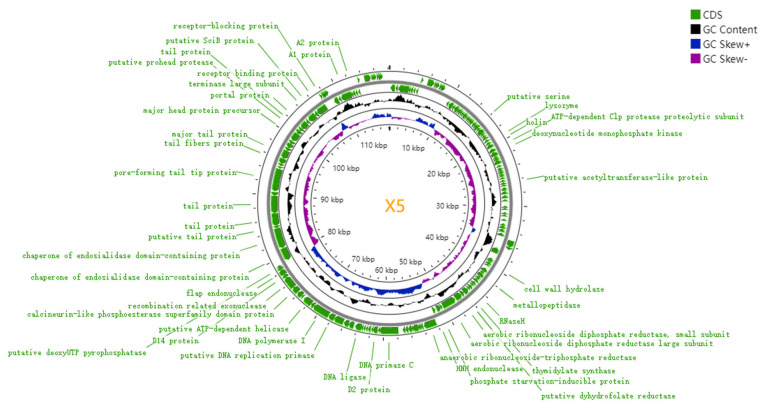
Genome map of phage X5 generated by CGView. Green areas indicate the distribution of the coding sequence (CDS) regions; arrows indicate the direction of transcription. The total GC.

The genome sequencing results for phage X5 are shown in Figure 3. The genome consists of 118,240 bp of double–stranded DNA with a GC content of 39.40%. The protein sequences of all genes were compared with those in CARD and VFDB database. No pathogenic factors, resistance genes, genes related to phage lysogenicity, or virulence genes were found, indicating their safety. BLAST–N analysis showed that the X5 genome had the highest homology with *Salmonella* phage S124 (GenBank: OK108607) of the Myoviridae family, with 98.51% homology.

**Figure 4:**
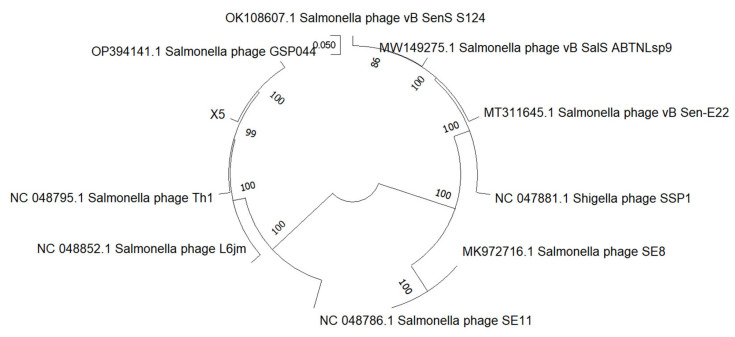
Phylogenetic evolutionary tree of phage X5. The phylogenetic tree was constructed based on the neighbor–joining method of the terminase large subunit.

Figure 4 shows the multi–genome alignment and phylogenetic tree analysis of phage X5 and *Salmonella* phage S124, ABTNLsp, E22, SSP1, SE8, SE11, GSP044, Th1, and L6jm.

**Figure 5:**
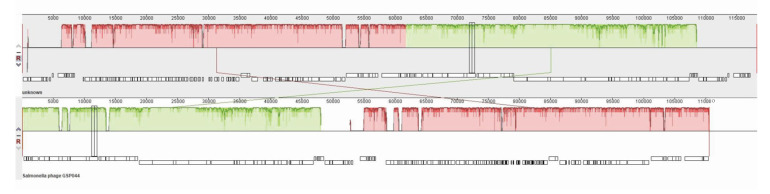
Collinearity analysis of phage X5 and GSP044 genomes.

GSP044 and X5 with the highest similarity were selected for genomic collinearity analysis. Some genes were in different regions, indicating that phage X5 may exhibit gene rearrangement (Figure 5). Similar color blocks represent putative homologous blocks. The height of the internal lines in the block represents the conservatism of the average sequence in the region, and the higher the height, the better the conservatism. The white area inside the block represents a region where homologous blocks cannot be found in other genomes. The top and bottom of the block represent the justice chain and the antisense chain. Red usually indicates a mismatch between two genomes, and green indicates a match. Phage GSP044 (GenBank: OP394141) was isolated from Huizhou City, Guangdong Province, China. 

Table 2 shows the protein analysis of phage X5. The phage genome consists of several gene cluster modules: DNA replication; repair and modification enzymes such as DNA helicase (ORF124), DNA replication primase (ORF121), and DNA polymerase (ORF122); DNA packaging proteins such as terminase small subunit (ORF95), portal protein (ORF150), and terminase large subunit (ORF152); lysis compounds such as lysozyme (ORF41) and holin (ORF42); and structural proteins such as tail fiber protein (ORF142), baseplate protein (ORF117), capsid protein (ORF147), and scaffold protein (ORF101). ORF100 encodes the phage principal protein. ORF99 encodes HNH endonuclease, an intron protein with sequence tolerance and site specificity. Thymidylate synthase (ORF93) is involved in nucleotide metabolism. The X5 genome encodes some additional proteins, such as serine/threonine phosphatase (ORF36) for amino acid biosynthesis. ATP-dependent helicase (ORF124) is involved in fundamental biological processes such as transcription, proliferation, and repair of DNA. ORF143 encodes the major tail protein of phage, while ORF142 encodes the tail fiber of phage. The phylogenetic tree of X5 was constructed based on the nucleotide sequence of the terminase large subunit (ORF152). The location and homology of the major ORFs in phage X5 and GSP044 are shown in Table 2. It is worth noting that the X5’s ORF99 does not exist in GSP044, which is thought to be a gene insertion. 

No phage transposase, toxins, excision enzyme homology, virulence factors, or repressors were predicted in the X5 genome. According to the sequencing results, phage X5 is a novel phage.

### 3.7. Scavenging Effect of Phage X5 on Bacterial Biofilm

**Figure 6:**
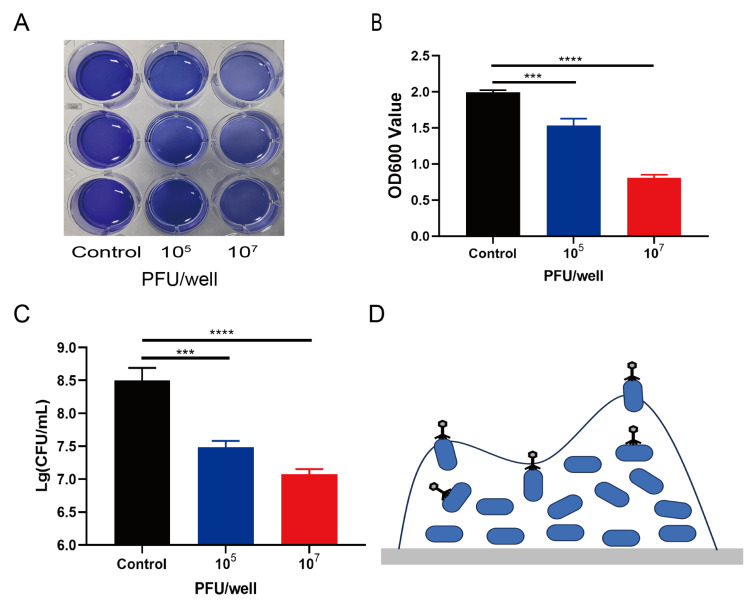
Effect of phage X5 on bacterial biofilm. The initial titers of phage X5 were 10^5^ and 10^7^ PFU/well. The biofilm biomass was obtained after incubation for 24 h. (**A**) Crystal violet staining analysis. (**B**) Optical density values measured at 600 nm. (**C**) Plate count results. The results are expressed as the mean ± SD (standard deviation) of three independent experiments. The one–way ANOVA method was used to assess significant differences between control and test samples. *** *p* < 0.001, **** *p* < 0.0001. (**D**) Pattern diagram of phage entry into bacteria.

To evaluate whether phage X5 can destroy the biofilm formed by *S. pullorum* 519, the biofilm treated with phage X5 was stained using the CV method. Phage X5 significantly removed the biofilm, even at low concentrations (10^5^ PFU/mL; Figure 6A,B (*p* < 0.001). Compared with the control group, the phage–treatment group had a significantly lower bacterial load (Figure 6C) (*p* < 0.001). This finding suggests that phage X5 can effectively reduce the biofilm formed by *S. pullorum* 519. Phage X5 effectively reduced the number of bacteria in the biofilm (Figure 6D).

### 3.8. Inactivation of Salmonella in Different Food Models by Phage X5

The bactericidal power of phage X5 was evaluated in solid foods (raw chicken) and liquid foods (milk).

#### 3.8.1. Bacteriostasis of Phages in Milk

**Figure 7:**
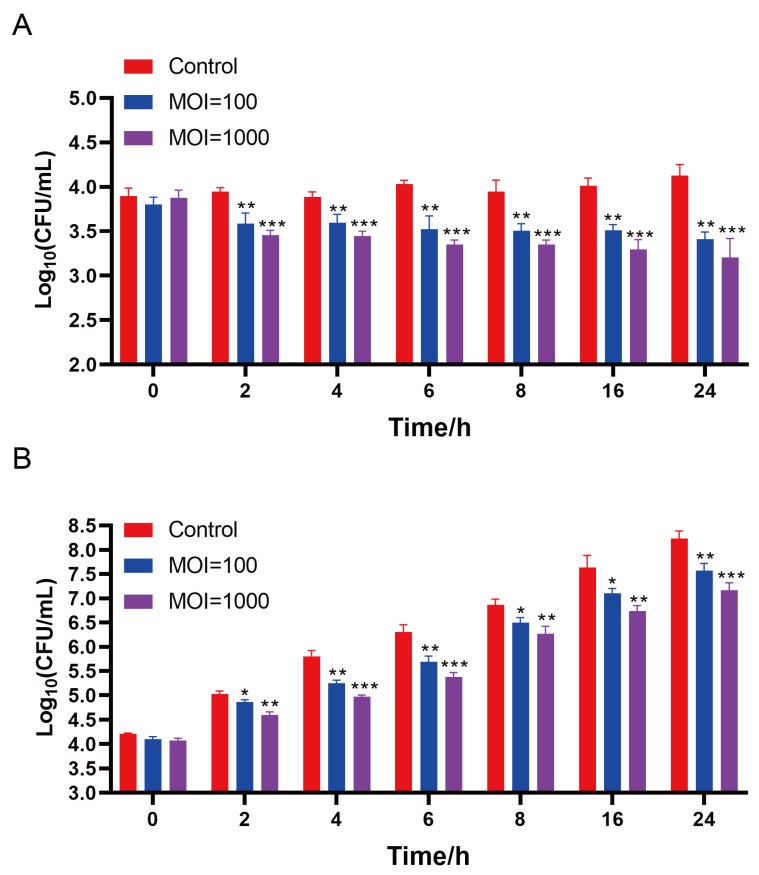
Application of phage X5 in the biological control of *S. pullorum* 519 in milk. (**A**) Effects of phage X5 on the growth of *S. pullorum* 519 in milk at 4 °C. (**B**) Effects of phage X5 on the growth of *S. pullorum* 519 in milk at 25 °C. * *p* < 0.05; ** *p* < 0.01; *** *p* < 0.001.

The inhibitory effect of phage X5 on *Salmonella* in milk is shown in Figure 7A.

Phage X5 at MOI of 100 and 1000 reduced live *Salmonella* counts (*S. pullorum*) compared with untreated controls. At 4 °C, the number of bacteria in the control group did not increase with time and remained at about 4 log_10_ CFU/mL. Following incubation at 4 °C for 24 h, compared to the control group, the number of viable bacteria in the MOI = 100 and MOI = 1000 groups were reduced by 0.72 log_10_ CFU/mL (*p* < 0.01) and 0.93 log_10_ CFU/mL (*p* < 0.001), respectively (Figure 6A). The maximum antibacterial efficiency was 80.70 and 88.10%, respectively.

At 25 °C, the inhibitory effect of phage X5 on *Salmonella* in milk was similar to that at 4 °C. The number of *Salmonella* in the control group increased significantly with prolonged time within 24 h, from 4.21 log_10_ CFU/mL to 8.23 log_10_ CFU/mL (Figure 7B). *Salmonella* counts were reduced by 0.66 log_10_ CFU/mL (*p* < 0.01) and 1.06 log_10_ CFU/mL (*p* < 0.001) with MOI of 100 and 1000, respectively. The inhibitory effect was greater at MOI = 1000 than at MOI = 100.

#### 3.8.2. Bacteriostasis of Phages in Chicken Breast

**Figure 8:**
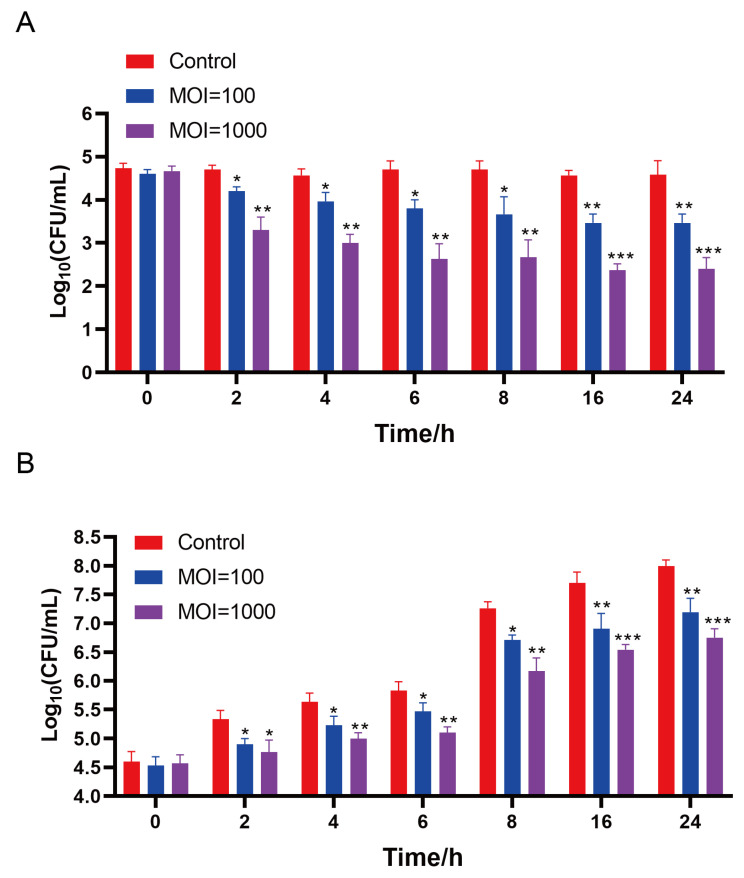
Application of phage X5 in biological control of *S. pullorum* 519 in chicken breast. (**A**) Effects of phage X5 on the growth of *S. pullorum* 519 in chicken breast at 4 °C. (**B**) Effects of phage X5 on the growth of *S. pullorum* 519 in chicken breast at 25 °C. * *p* < 0.05; ** *p* < 0.01; *** *p* < 0.001.

The inhibitory effect of phage X5 on host bacteria on chicken surface is shown in Figure 8. At 4 °C, at 2–24 h phage treatment with an MOI of 100, the *Salmonella* count on chicken breast meat decreased from 4.6 log_10_ CFU/mL to 3.47 log_10_ CFU/mL (*p* < 0.01; Figure 8A). After treatment with a MOI of 1000 for 2–24 h, the *Salmonella* count on chicken breast decreased from 4.6 log_10_ CFU/mL to 2.18 log_10_ CFU/mL (*p* < 0.001) (Figure 8B). At 25 °C, the total number of *Salmonella* bacteria in each group gradually increased with time. At 2 h, the number of *Salmonella* counts was significantly lower in the phage–treated groups (MOI = 100 and 1000) than in the control group. At 24 h, the total amount of *Salmonella* bacteria in the phage–treated groups decreased by 0.81 log_10_ CFU/mL (MOI = 100) and 1.25 log_10_ CFU/mL (MOI = 1000) compared with the control group. The results showed that phage X5 had an adequate bacteriostatic effect on the chicken surface. Additionally, the bacteriostatic effect was greater at MOI = 1000 than at MOI = 100.

## 4. Discussion

Phages can efficiently lysate pathogenic bacteria. The application of phages reduces the need for antibiotics and promotes food safety. Phages have been widely used in clinical medicine, animal husbandry, aquaculture, and agriculture [3,42].

*S. pullorum* causes *pullorum* disease, which persists in adult chickens without obvious clinical symptoms and can spread vertically and horizontally, endangering the development of the poultry industry [43,44]. *Salmonella* phage CKT1 effectively controls the vertical transmission of *S. pullorum* in adult broilers [45]. Specifically, phage CKT1 significantly reduces weight loss in chickens infected with *S. pullorum* by regulating the abnormal intestinal flora and can be used as a potential substitute for antibacterial growth promoters in poultry farms [46]. Controlling *S. pullorum* in chicken farms is an effective strategy to prevent human infection.

The application of phages for the control of bacterial pathogens in foods is an emerging area. The commercial interest in this area has gradually increased since the FDA approved the limited use of phages in fresh foods to control *Listeria monocytogenes* [47]. Phages have host specificity, which is an important factor limiting the therapeutic efficacy of phages against bacterial infections [48]. Therefore, phages with broad host spectrum have broad application prospects. A broad–spectrum *Salmonella* phage capable of lysing five *Salmonella* serotypes has been isolated [49].

As research advances, the issues of phage safety, stability, and the development of bacterial resistance will be resolved, and the screening of novel phages and mixed preparations will become a hot spot in the antibacterial realm [50]. The latency of phage X5 was shorter than those of other phages reported, which may be related to the high lytic activity of phage X5 [51,52]. Phages with short latency can lyse more bacteria in a short period of time; hence, they are suitable for biological control [53,54].

Phage X5 was stable at pH 4–12 and had high pH tolerance. The stability of phage X5 in acidic and alkaline conditions allows it to be used in food with different pH values. Phage X5 had better bacteriostatic effects in milk than in chicken breast, which may be due to the adsorption of phage to bacteria in a liquid food matrix as result of the fluidity of milk [55]. Phage X5 has a binary lysis system composed of holin and lysozyme, which destroy the cell membrane and cell wall of bacteria, respectively, and has a completely different mechanism of action from antibiotics [56,57]. The tail fiber protein is responsible for the specific initial recognition of host bacteria and can be a potential biological cognitive element for detecting bacteria [58,59]. The application of phages and their derivatives, such as endolysin, as biological control agents has been documented [60].

In the study, the genomic and biological characteristics of the isolated phage X5 were evaluated. Phage X5 has a broad host range, high tolerance to extreme conditions, and strong antibacterial ability. The lysis rate of phage X5 was 87.50% (70/80), which was higher than that of phage GSP044, at 81.25% (39/48) [61]. For phage-insensitive *Salmonella* strains, it is speculated that the tail protein of phage X5 cannot recognize these bacteria. The specific recognition mechanism or the use of phage cocktails will be investigated in the future [62]. Additionally, phage X5 had an adequate scavenging effect on the mature bacterial biofilm and effectively reduced the bacterial load in the biofilm. Compared with plant essential oils, enzyme preparation, and lactic acid bacteria, although they have antibacterial effects, their antibacterial spectrum is relatively wide, which may affect or destroy the balance of normal flora. Phages have the advantages of strict host specificity, strong exponential proliferation ability as well as high safety and ecological friendliness in the field of anti–pathogenic biofilms, which gives phages broad application prospects in the field of antimicrobial therapy [63]. The bacterial load was significantly lower in the treatment groups (MOI of 100 and 1000) than in the control group at 24 h, indicating that phage X5 could significantly inhibit the host bacteria in the milk matrix, whether at 4 °C or 25 °C. These findings suggest that phage X5 is a promising and effective biological control agent for controlling *Salmonella* infection in the food industry. At present, we have studied the recognition receptor of phage X5, preliminarily analyzed the receptor site, and studied the phage resistance.

The application of phages in the food industry has considerable potential but also faces some challenges and comes with potential risks, such as consumer acceptance, security assessment, technical limitations, potential drug resistance, and effective use in food production. Therefore, there are no approved phage food bacteriostatic agents in China. However, the phage product of Qingdao Phagepharm Bio–Tech Co., Ltd. (Qingdao, China) has been approved by The U. S. Food and Drug Administration (FDA). Considered Generally Recognized as Safe (GRAS), this phage product is expected to play an important role in the international market (https://www.fda.gov/media/178868/download, accessed on 30 May 2024). Our future work will focus on the prevention of *Salmonella* infection by X5 lyase and on the identification of receptor–binding proteins. Phage X5 has the potential to be used in phage preparations for the prevention of *Salmonella* infection in the food, agriculture, medical, aquaculture, livestock, agroforestry, and environmental industries.

## 5. Conclusions

A virulent phage X5 that targets *S. pullorum* was isolated in this study. Phage X5 exhibits a broad host range, resistance to heat and pH variations, and a short latency period. The results revealed that phage X5 has potential application value as a biocontrol agent for removing biofilm formation of *S. pullorum* and as an alternative for the prevention and control of *S. pullorum* contamination of milk and chicken meat.

## Figures and Tables

**Table 1:** Lytic activity of phage X5 against the tested strains.

Strains	X5 ^a^	Strains	X5 ^a^	Strains	X5 ^a^
*S. pullorum* CVCC529	+	*S. typhimurium* ATCC14028	+	*S. typhimurium* 3	+
*S. pullorum* CVCC530	+	*S. typhimurium* CMCC50115	+	*S. typhimurium* 129	+
*S. pullorum* CVCC531	+	*S. typhimurium* SL1344	+	*S. typhimurium* 107	+
*S. pullorum* CVCC534	+	*S. typhimurium* 1	+	*S. typhimurium* 6	+
*S. pullorum* CVCC535	+	*S*. *typhimurium* 10	+	*S. typhimurium* 8	+
*S. pullorum* CVCC540	+	*S. typhimurium* 11	+	*S. typhimurium* ST24	−
*S. pullorum* 1	+	*S. typhimurium* 16	+	*S. typhimurium* 27	+
*S. pullorum* 4	−	*S. typhimurium* 17	−	*S. typhimurium* 130	+
*S. pullorum* 5	+	*S. typhimurium* 19	+	*S. typhimurium* 5	+
*S. pullorum* 6	+	*S. typhimurium* 20	+	*S. typhimurium* 78	−
*S. pullorum* 12	+	*S. typhimurium* 93	+	*S. typhimurium* G7	+
*S. pullorum* 16	+	*S. typhimurium* 86	+	*S. typhimurium* 74	+
*S. pullorum* 59	+	*S. typhimurium* 90	+	*S. typhimurium* P17	+
*S. pullorum* 64	−	*S. typhimurium* 77	+	*S. typhimurium* 21	+
*S. pullorum* 78	+	*S. typhimurium* P6	+	*S. typhimurium* G20	−
*S. pullorum* 84	+	*S. typhimurium* 74	+	*S. typhimurium* G10	+
*S. pullorum* 85	+	*S. typhimurium* 80	+	*S. typhimurium* G7	+
*S. pullorum* 92	+	*S. typhimurium* 85	+	*S. typhimurium* G1	+
*S. pullorum* 104	+	*S. typhimurium* 83	+	*S. typhimurium* 24	+
*S. pullorum* ATCC9120	−	*S. typhimurium* ST1	+	*S. typhimurium* ST21	+
*S. pullorum* 127	+	*S. typhimurium* 87	−	*S. typhimurium* P11	+
*S. pullorum* 153	+	*S. typhimurium* 7	+	*S. typhimurium* 88	+
*S. enteritidis* CVCC3375	+	*S. typhimurium* 81	+	*S. typhimurium* ST22	+
*S. enteritidis* CMCC50746	+	*S. typhimurium* 11	+	*S. typhimurium* ST25	−
*S. enteritidis* 7	−	*S. typhimurium* 82	+	*L. acidophilus* ATCC832	−
*S. enteritidis 23*	+	*S. typhimurium* P8	+	*C. perfringensa* ATCC13124	−
*S. enteritidis25*	+	*S. typhimurium* 79	+	*K. pneumoniae* Y1	−
*S. enteritidis 61*	−	*S. typhimurium* 180	+	*E. coli* ATCC25922	

^a^ Symbols: After the bacteria to be tested are infected with phage X5, the (+) region is clear or the (−) region has no plaques. CMCC: National Center for Medical Culture Collections. ATCC: American Type Culture Collection. The other strains tested were clinical isolates.

**Table 2:** Functional analysis of major proteins of phage X5.

X5					GSP044			Function
ORF	Strand	Start	Stop	Strand	Start	Stop	Homology	
ORF3	−	1383	3137	−	49,965	51,720	92%	A1 protein
ORF5	−	3393	3800	−	51,976	52,383	99%	A2 protein
ORF34	−	15,682	16,272	−	65,190	65,778	96%	putative serine/threonine protein phosphatase
ORF36	−	16,561	17,424	−	66,067	66,930	94%	serine/threonine protein phosphatase
ORF41	−	19,005	19,468	−	68,561	68,974	98%	lysozyme
ORF42	−	19,465	20,121	−	68,971	69,627	99%	holin
ORF43	−	20,278	20,877	−	69,784	70,383	99%	ATP-dependent Clp protease proteolytic subunit
ORF44	−	20,890	21,642	−	70,396	71,148	98%	deoxynucleotide monophosphate kinase
ORF56	−	26,089	26,457	−	75,595	75,963	99%	putative acetyltransferase-like protein
ORF91	−	44,339	44,815	−	93,911	94,385	97%	RNaseH
ORF93	−	45,190	46,044	−	94,760	95,614	97%	thymidylate synthase
ORF94	−	46,044	46,577	−	95,614	96,147	98%	putative dyhydrofolate reductase
ORF95	−	46,574	47,719	−	96,144	97,289	96%	aerobic ribonucleoside diphosphate reductase, small subunit
ORF96	−	47,826	50,156	−	97,396	99,726	98%	aerobic ribonucleoside diphosphate reductase large subunit
ORF98	−	50,522	51,274	−	100,093	110,563	99%	phosphate starvation-inducible protein
ORF99	−	51,460	51,951					HNH endonuclease
ORF100	+	52,145	54,019	+	101,200	103,074	97%	anaerobic ribonucleoside-triphosphate reductase
ORF109	+	57,833	60,622	+	106,613	109,402	97%	DNA primase C
ORF111	+	60,908	61,612	+	109,688	110,392	97%	D2 protein
ORF117	+	63,312	64,283	+	1606	2577	98%	DNA ligase
ORF118	+	64,486	65,265	+	2780	3559	98%	DNA ligase
ORF121	+	67,577	68,467	+	6401	7291	97%	putative DNA replication primase
ORF122	+	68,530	71,097	+	7741	10,308	96%	DNA polymerase I
ORF124	+	71,584	72,936	+	10,795	12,147	97%	putative ATP-dependent helicase
ORF127	+	74,244	75,221	+	13,967	14,944	95%	calcineurin-like phosphoesterase superfamily domain protein
ORF128	+	75,202	77,040	+	14,925	16,763	97%	recombination related exonuclease
ORF129	+	77,044	77,526	+	16,767	17,249	98%	D14 protein
ORF130	+	77,526	78,401	+	17,249	18,124	98%	flap endonuclease
ORF131	+	78,398	78,844	+	18,121	18,567	95%	putative deoxyUTP pyrophosphatase
ORF133	−	79,111	81,198	−	18,837	20,924	96%	chaperone of endosialidase domain-containing protein
ORF134	−	81,241	84,591	−	20,967	24,317	99%	chaperone of endosialidase domain-containing protein
ORF135	−	84,591	85,013	−	24,317	24,739	100%	putative tail protein
ORF136	−	85,018	87,075	−	24,744	26,801	99%	tail protein
ORF137	−	87,075	89,924	−	26,801	29,650	98%	tail protein Pb3
ORF139	−	90,645	94,352	−	30,371	32,567	94%	pore-forming tail tip protein
ORF142	−	95,267	96,166	−	34,966	35,865	98%	tail fibers protein
ORF143	−	96,171	97,577	−	35,877	37,279	93%	major tail protein
ORF147	−	99,432	100,808	−	39,134	40,510	92%	major head protein precursor
ORF148	−	100,826	101,458	−	40,528	41,160	99%	putative prohead protease
ORF149	−	101,462	101,953	−	41,315	41,646	86%	tail protein
ORF150	−	101,950	103,167	−	42,082	42,558	99%	portal protein
ORF152	−	103,719	105,035	−	43,258	44,574	94%	terminase large subunit
ORF153	−	105,035	105,517	−	44,574	45,056	98%	putative SciB protein
ORF154	−	105,528	107,315	−	45,067	46,854	97%	receptor binding protein
ORF155	+	107,401	107,667	+	46,940	47,206	100%	receptor-blocking protein
ORF163	−	110,115	111,869	−	49,965	51,720	92%	A1 protein
ORF165	−	112,125	112,532	−	51,976	52,383	99%	A2 protein

## Data Availability

The original contributions presented in the study are included in the article, further inquiries can be directed to the corresponding authors.
